# A preclinical model of glioblastoma

**Published:** 2015-01

Gliobastoma multiforme (GBM) is an aggressive malignant form of brain tumour for which effective therapies are limited. Genetically engineered mouse models (GEMMs) harbouring mutations in components of the main signalling pathways that are altered in human GBM – including the receptor tyrosine kinase (RTK)–RAS– phosphoinositide-3-kinase (PI3K) pathway – are available. However, these GEMMs display a long latency to tumorigenesis and advanced tumour heterogeneity, which represents a challenge in preclinical drug testing. In this study, Zoë Weaver Ohler and colleagues developed an orthotopic and tractable mouse model of GBM by transplanting brain tumour cells derived from GBM-GEMMs into the brain (orthotopically) of syngeneic mice (non-transgenic wild-type mice with identical genetic backgrounds and intact immune systems). This model develops GBM with features of the human disease, including high vascularisation and aggressive invasion of surrounding tissues, and thus was used to test the effects of two drugs that are currently in clinical trials for GBM and other solid tumours, namely BKM120 and PD0325901 [inhibitors of PI3K and mitogen-activated protein kinase (MAPK), respectively]. When tested *in vitro* and *in vivo* as single agents, neither drug improved mouse survival. However, combination therapy increased cancer cell apoptosis, decreased tumour cell proliferation and enhanced survival, owing to a synergistic effect of the drugs on suppression of the PI3K pathway (which regulates cell proliferation and survival). Therefore, this model represents a valuable preclinical system for advancing current therapies and for testing novel drugs and drug combinations against GBM. **Page 45**

**Figure:**
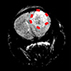


